# Ginseng Berry Extract Promotes Maturation of Mouse Dendritic Cells

**DOI:** 10.1371/journal.pone.0130926

**Published:** 2015-06-19

**Authors:** Wei Zhang, Si-Young Cho, Gao Xiang, Kyung-Jin Min, Qing Yu, Jun-O Jin

**Affiliations:** 1 Shanghai Public Health Clinical Center, Shanghai Medical College, Fudan University, Shanghai, China; 2 R&D Unit, AmorePacific Corporation, 1920 Yonggudae-ro, Giheung-gu, Yongin-si, Gyeonggi-do, Korea; 3 Model Animal Research Center, Nanjing University, Nanjing, China; 4 Department of Biological Sciences, Inha University, Incheon, Korea; 5 Department of Immunology and Infectious Diseases, The Forsyth Institute, 245 First Street, Cambridge, MA, United States of America; Istituto Superiore di Sanità, ITALY

## Abstract

Ginseng extract has been shown to possess certain anti-virus, anti-tumor and immune-activating effects. However, the immunostimulatory effect of ginseng berry extract (GB) has been less well characterized. In this study, we investigated the effect of GB on the activation of mouse dendritic cells (DCs) *in vitro* and *in vivo*. GB treatment induced up-regulation of co-stimulatory molecules in bone marrow-derived DCs (BMDCs). Interestingly, GB induced a higher degree of co-stimulatory molecule up-regulation than ginseng root extract (GR) at the same concentrations. Moreover, *in vivo* administration of GB promoted up-regulation of CD86, MHC class I and MHC class II and production of IL-6, IL-12 and TNF-α in spleen DCs. GB also promoted the generation of Th1 and Tc1 cells. Furthermore, Toll like receptor 4 (TLR4) and myeloid differentiation primary response 88 (MyD88) signaling pathway were essential for DC activation induced by GB. In addition, GB strongly prompted the proliferation of ovalbumin (OVA)-specific CD4 and CD8 T cells. Finally, GB induced DC activation in tumor-bearing mice and the combination of OVA and GB treatment inhibited B16-OVA tumor cell growth in C57BL/6 mice. These results demonstrate that GB is a novel tumor therapeutic vaccine adjuvant by promoting DC and T cell activation.

## Introduction

A recent effort in the development of new medication and immunomodulatory agents is to search for candidates among natural products, since they have relatively low toxicities in clinical applications [1]. Ginseng root (GR) has been used in Korean, Japan and China as a traditional medicine and has demonstrated efficacy against various human diseases, such as cancer, viral infectious diseases, diabetes, and atherosclerosis [2]. The reports from early studies showed that GR has strong immunostimulatory properties, such as modulating macrophage and dendritic cell (DC) activation, proliferation and viability of mouse spleen cells [3, 4]. Recent studies demonstrated that in addition to GR, ginseng berries (GB) and leaves also have immunostimulatory effect [5–7]. However, *in vivo* immune activation effects of GB, especially those on DC and T cell activation and anti-cancer immune responses, have not been investigated.

DCs are professional antigen presenting cells (APCs) and key modulators of adaptive immunity mainly owing to their superior ability to take up and present antigens (Ags) [8, 9]. DCs exist in two functionally and phenotypically distinct stages, immature and mature DCs. Upon exposure to microbial stimuli or Ags, immature DCs phagocytose Ags and undergo a maturation process characterized by increased expression of co-stimulatory molecules, production of pro-inflammatory cytokines and presentation of Ags to T cells [8–10]. Moreover, DCs have two main modes of antigen presentation, which are cross-present of exogenous Ags through MHC class I to CD8 T cells and direct-presentation of extracellular captured Ags through MHC class II to CD4 T cells [11, 12].

Ags used for vaccine are often poorly immunogenic and require additional reagents, termed adjuvants, to enhance the induction of Ag-specific immune responses, as indicated by antibody production and effector T cell functions [13, 14]. Since tumor vaccine seeks to induce CD8 T cell activation against tumor Ags, DC-mediated cross-presentation of tumor Ags is required [8, 14] To promote activation of CD8 T cells, an effective adjuvant must be given together with Ag peptide, formulated in a manner that facilitates entry of Ags into the MHC class I processing pathway, trigger DC activation and maturation.

The present study is undertaken to test that *in vivo* administration of GB can induce the activation of spleen DCs and the consequent activation and proliferation of Ag-specific T cells and anti-tumor response, which may serve as a potential new vaccine adjuvant to combat viral and bacterial infection and cancer.

## Materials and Methods

### Mice and cell lines

C57BL/6 mice (6 weeks old) were purchased from the B&K Laboratory Animal Corp (Shanghai). TLR2^-/-^ and TLR4^-/-^ were obtained from Nanjing Animal Center. MyD88^-/-^, OT-I and OT-II TCR transgenic mice and C57BL/6-Ly5.1 (CD45.1) congenic mice were obtained from Shanghai Public Health Clinical Center, and kept under pathogen-free conditions. All experiments were carried out under the guidelines of the Institutional Animal Care and Use committee at the Shanghai Public Health Clinical Center. The protocol was approved by the committee on the Ethics of Animal Experiments of the Shanghai Public Health Clinical Center (Mouse Protocol Number: SYXK-2010-0098). The murine melanoma cell line B16F10 (ATCC, CRL-6475) expressing OVA (B16-OVA) was cultured in 10% FCS RPMI (Sigma Aldrich, 2 mM glutamine, 1 M HEPES, 100 μg/ml streptomycin and 100 U/ml penicillin, 2 mM 2-mercaptoethanol). All cell lines were cultured at 37°C in a humidified atmosphere of 5% CO_2_ and air.

### Chemicals and cytokines

GR was obtained from Korea Ginseng Corporation (Seoul, Republic of Korea) and GB was provided from R&D Center, Amorepacific Corporation as described previously (Gyeongi-do, Republic of Korea) [15]. Chicken ovalbumin (OVA) was obtained from Sigma-Aldrich. The endotoxin levels contained in the amount of GR and GB (50 mg/kg) and OVA (50 μg) used in each *in vivo* experiment were evaluated using a Limulus amebocyte lysate (LAL) assay kit (Lonza) and were less than 0.1 endotoxin unit/ml.

### Antibodies

Isotype control antibodies (Abs) (IgG1, IgG2a or IgG2b), CD11c (HL3), CD4 (GK1.5), CD8α (YTS169.4), CD40 (3/23), CD80 (16-10A1), CD86 (GL-1), anti-IL-4 (11B11), anti-IL-6 (MP5-20F3) and anti-IL-12/23p40 (C17.8) were from BioLegend; anti-MHC class I (AF6-88.5.3), anti-MHC class II (M5/114.15.2), anti-IFN-γ (XMG1.2), anti-IL-17 (TCC11-18H10.1) and anti-TNF-α (MP6-XT22) were from eBioscience.

### Flow cytometry analysis

Cells were washed with phosphate buffered saline (PBS) (Gibco) containing 0.5% BSA (Sigma-Aldrich), pre-incubated for 15 min with unlabeled isotype control Abs, and then labeled with fluorescence-conjugated Abs by incubation on ice for 30 min followed by washing with PBS. Cells were analyzed on a FACS Aria II (Becton Dickinson) and FlowJo 8.6 software (Tree Star). Cellular debris was excluded from the analysis by forward- and side-scatter gating. Dead cells were further excluded by 7 aminoactinomycin D (7AAD) (BioLegend) staining and gating on the 7AAD-negative population. As a control for nonspecific staining, isotype-matched irrelevant mAbs were used.

### *In vitro* BMDC generation

The initial cultures were prepared as described elsewhere [16]. Bone marrow nucleated cells (1 × 10^6^ cells/ml) were cultured in modified 5 ml RPMI 1640 (Gibco) medium containing 10% FBS (Gibco), in 6 well plates. 50 ng/ml rmGM-CSF (Peprotech) plus 50 ng/ml rmIL-4 (Peprotech) were added in the medium for BMDC. Unless otherwise stated, cells were cultured normally for 6 days at 37°C in 10% CO_2_ in air. The cultured cells were washed twice in fresh medium before additional experiments.

### Spleen DC analysis

Spleens were cut into small fragments and digested, with 2% fetal bovine serum (FCS) containing collagenase IV (Gibco) for 20 min at room temperature. Cells from the digest were centrifuged and the cell pellet was resuspended in 5 mL of 1077 histopaque (Sigma-Aldrich). More histopaque was then layered below the cell suspension, with EDTA-FCS-layered above it. After centrifugation at 1700g for 10 min, the light density fraction (< 1.077 g/cm3) was collected and incubated for 30 min with the following FITC-conjugated monoclonal antibodies (mAbs): anti-CD3 (17A2), anti-Thy1.1 (OX-7), anti-B220 (RA3-6B2), anti-Gr1 (RB68C5), anti-CD49b (DX5) and anti-TER-119 (TER-119). Cells were analyzed on a FACS Aria II (Becton Dickinson). The spleen DCs were identified as lineage^-^CD11c^+^ cells.

### *Ex vivo* cell stimulation and intracellular cytokine staining

Singles cells prepared from spleens were stimulated *in vitro* for 4 hours with phorbol 12-myristate 13-acetate (50 ng/ml) and ionomycin (1 μM; both from Calbiochem), with the addition of monensin solution (2 μM; Biolegend) during the final 2 hours. For intracellular cytokine staining, cells were stained for surface molecules first, then fixed and permeabilized with Cytofix/Cytoperm buffer (eBioscience) and subsequently incubated with anti-cytokine antibodies in Perm/Wash buffer (eBioscience) for 30 min. Control staining with isotype control IgGs was performed in all experiments.

### ELISA

IL-6, IL-12p70, and TNF-α concentrations in the sera were measured in triplicate using standard ELISA kits (Biolegend).

### Real-time qPCR

Total RNA was reverse-transcribed into cDNA using Oligo (dT) and M-MLV reverse transcriptase (Promega). The cDNA was subjected to real-time PCR amplification (Qiagen) for 40 cycles with annealing and extension temperature at 60°C, on a LightCycler 480 Real-Time PCR System (Roche). Primer sequences are: mouse β-Actin forward, 5’-TGGATGACGATATCGCTGCG-3’; reverse, 5’-AGGGTCAGGATACCTCTCTT-3’, IL-6 forward, 5’-AACGATGATGCACTTGCAGA-3’; reverse, 5’-GAGCATTGGAAATTGGGGTA-3', IL-12p40 forward, 5’-CACATCTGCTGCTCCACAAG-3’; reverse, 5’- CCGTCCGGAGTAATTTGGTG-3’, TNF-α forward, 5’-CCTTTCACTCACTGGCCCAA-3’; reverse, 5’-AGTGCCTCTTCTGCCAGTTC-3’ T-bet forward, 5’-CAACAACCCCTTTGCCAAAG-3’; reverse, 5’-TCCCCCAAGCATTGACAGT-3’, GATA3 forward, 5’-CGGGTTCGGATGTAAGTCGAGG-3’; reverse, 5’- GATGTCCCTGCTCTCCTTGCTG-3’, RORγt forward, 5’-CCGCTGAGAGGGCTTCAC-3’; reverse 5’-TGCAGGAGTAGGCCACATTACA-3’, IFN-γ forward, 5’-GGATGCATTCATGAGTATTGC-3’; reverse, 5’-CTTTTCCGCTTCCTGAGG-3’, IL-4 forward, 5’-ACAGGAGAAGGGACGCCAT-3’; reverse 5’-GAAGCCCTACAGACGAGCTCA-3’, IL-17A forward, 5’-GCGCAAAAGTGAGCTCCAGA-3’; reverse 5’-ACAGAGGGATATCTATCAGGG-3’.

### OT-I and OT-II T cell proliferation

CD4 T cells from OT-II mice or CD8 T cells from OT-I mice were isolated from spleens using CD4 T cell or CD8 T cell isolation kit (Miltenyi Biotec), respectively. The cells were suspended in PBS/0.1% BSA containing 10 μM CFSE (Invitrogen) for 10 min. CFSE-labeled cells (1 × 10^6^) were *i*.*v*. transferred into CD45.1 congenic mice, and 24 hours later, mice were injected with PBS alone, 50 μg of OVA in PBS or OVA plus GB (50 mg/kg) in PBS. At 3 days after immunization, splenocytes were harvested and OT-I or OT-II T cell proliferation was determined by analyzing the CFSE fluorescence intensity through flow cytometry.

### DC activation in tumor and tumor protection

B16 melanoma cells (1 × 10^6^) were injected subcutaneously (*s*.*c*.) at the right flank of mice. 15 days later, mice with well-established tumor received GB administration and analyzed for DC activation in the spleen and tumor drLN. For the assessment of tumor protection, C57BL/6 mice were injected s.*c*. with B16-OVA melanoma cells (1 × 10^6^). Once the tumors were well established on day 7, mice received *i*.*p*. injection of PBS, 50 μg of OVA, 50 mg/kg of GB or the combination of OVA and GB. 5 days later, mice were treated with the same amount of OVA and GB again and the tumor growth was measured. Tumor size was measured every 5 days till day 22 after the initial tumor challenge.

### Statistical analysis

Results are expressed as the mean ± standard error of the mean (SEM). Statistical significance was determined by Student’s *t*-test (two-tailed, two-sample equal variance). P values smaller than 0.05 were considered as statistically significant.

## Results

### GB Promotes the Activation of BMDCs

**Fig 1:**
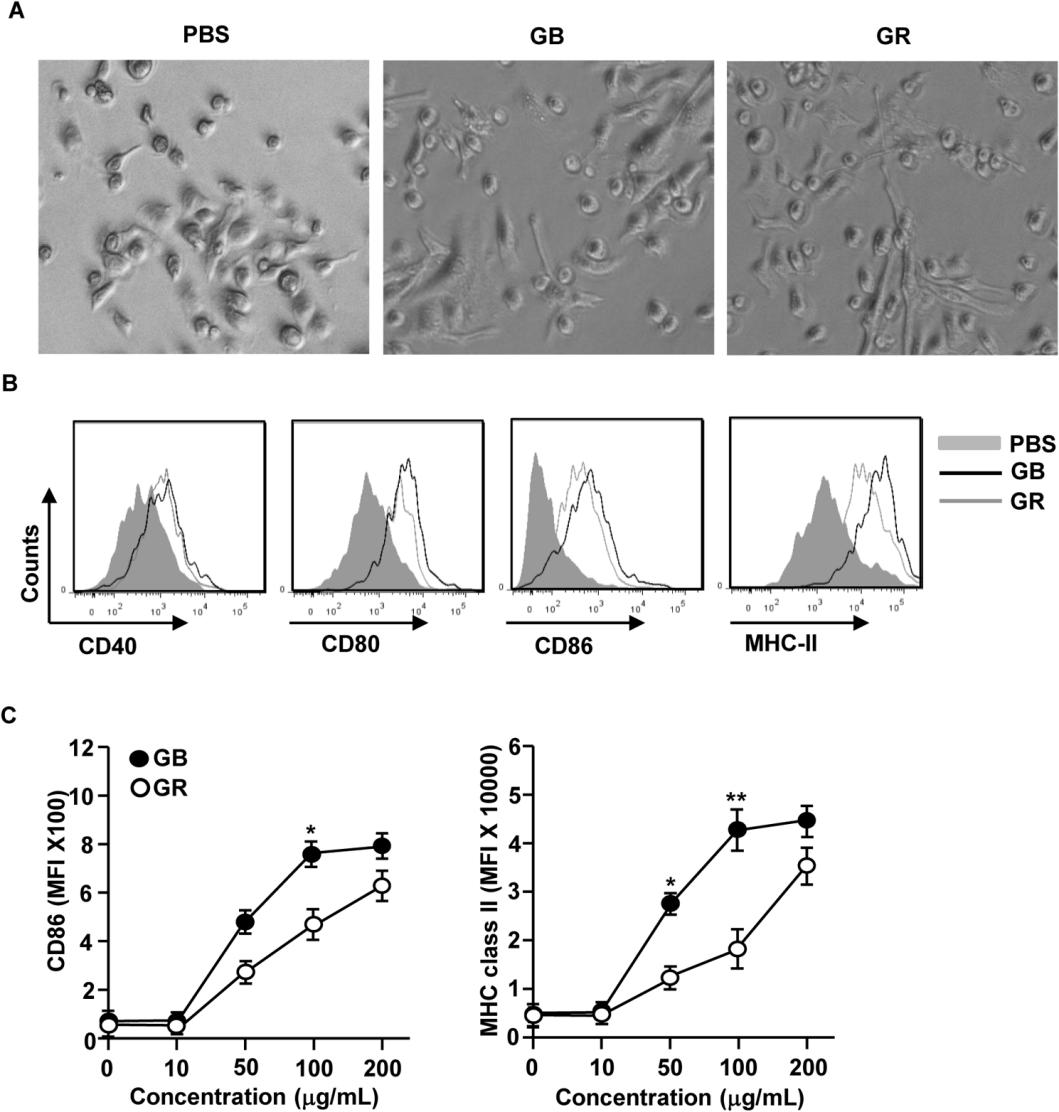
Activation of BMDC by GB. Bone marrow cells (1 X 10^6^) were incubated with 50 ng/ml GM-CSF and 50 ng/ml IL-4 for 6 days, and then stimulated with GB or GR for 24 hours. (A) Morphological changes were analyzed by microscopy. (B) Expression of surface co-stimulatory molecules measured by flow cytometry. (C) CD86 and MHC class II expression levels were measured from GR- or GB-treated BMDC in indicated dose. All data are representative of or the average of analyses of 6 independent samples (2 samples per experiment, total 3 independent experiments). *p < 0.*05*, **p < 0.*01*.

Previous reports showed that GR can induce the activation of mouse bone marrow-derived DCs (BMDCs) [17]. We therefore assessed whether GB can also induce the activation or maturation of BMDCs *in vitro*. Bone marrow cells were isolated from C57BL/6 mice and cultured with GM-CSF and IL-4 to generate immature BMDCs. After 6 days of culture, the majority of the cells were immature BMDCs based on the expression of CD11c. We further stimulated these cells with 100 μg/ml GB or GR, with the latter as a positive control. After 24 hours of culture, we found that treatment with GB promoted the dendritic morphological changes in BMDCs (Fig 1A). Moreover, the expression levels of co-stimulatory molecules CD40, CD80, CD86 and MHC class II on BMDCs were substantially increased by GB (Fig 1B). Interestingly, GB treatment showed a stronger effect on inducing BMDC activation than GR, as indicated by higher levels of co-stimulatory molecule expression in the BMDCs (Fig 1B). To confirm this observation, we assessed the dose-dependent effect of GB and GR on BMDC activation. GB treatment at 50 or 100 μg/ml induced more potent up-regulation of CD86 and MHC class II compared to GR at the same doses (Fig 1C). These data demonstrate that GB induces activation of BMDCs and its effect is stronger than that of GR.

### GB Induces the Activation of Spleen DCs *in vivo*

**Fig 2:**
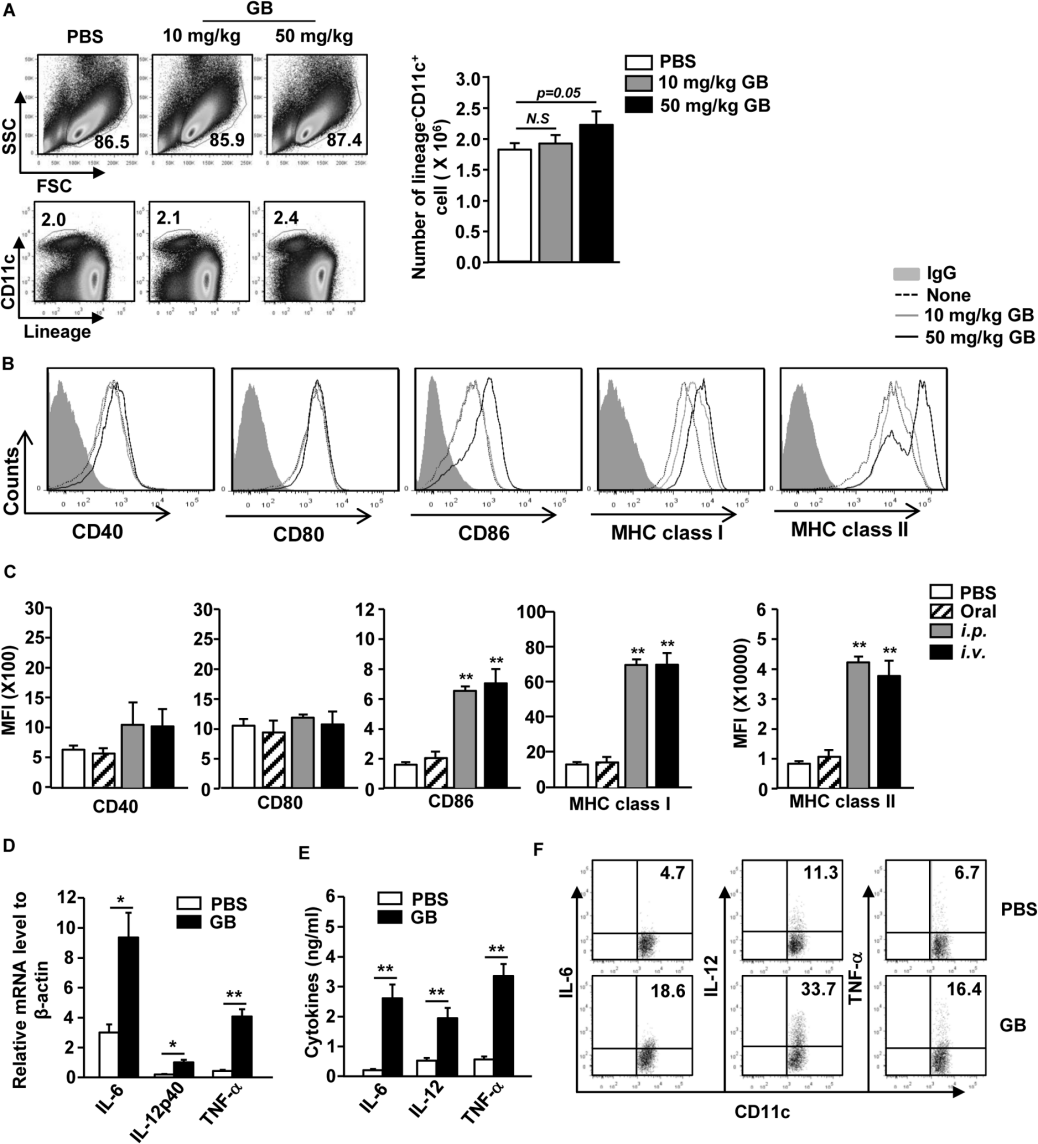
*In vivo* administration of GB induces spleen DC activation. C57BL/6 mice were injected *i*.*p*. with 10 or 50 mg/kg GB for 24 hours. (A) Percentage of lineage^-^CD11c^+^ spleen DCs was analyzed on a flow cytometry (left panels). Absolute cell number of lineage^-^CD11c^+^ cells within live cells were shown (right panel). (B) Expression levels of CD40, CD80, CD86, MHC class I and MHC class II were measured by flow cytometry. Data are representative of analyses of 8 independent samples. (C) C57BL/6 mice were treated orally, *i*.*p*. or *i*.*v*. with 50 mg/kg GB for 24 hours and measured Expression levels of CD40, CD80, CD86, MHC class I and MHC class II by flow cytometry. (D) Expression levels of IL-6, IL-12p40 and TNF-α mRNA were measured from spleen 2 hours after 50 mg/kg GB injection. (E) IL-6, IL-12p70 and TNF-α levels in sera were shown from spleen 24 hours after 50 mg/kg GB injection. (F) Intracellular cytokine production levels were measured spleen DCs. All data are from analyses of 6 individual mice each group (2 mice per experiment, total 3 independent experiments). *p < 0.*05*, **p < 0.*01*.

Our *in vitro* observation that GB promotes BMDC activation prompted us to examine the effect of GB on spleen DC activation *in vivo*. We injected 10 or 50 mg/kg GB intraperitoneally (*i*.*p*.*)* to C57BL/6 mice and analyzed spleen DCs 24 hours later. 50 mg/kg GB treatment led to a significant increase in the proportion and number of spleen DCs, which were identified as lineage^-^CD11c^+^ cells, whereas 10 mg/kg GB had no significant effect (Fig 2A, *p = 0*.*05*). Moreover, administration of 50 mg/kg GB induced a substantial increase in the surface levels of CD86, MHC class I and MHC class II in spleen DCs (Fig 2B). We also examined how the methods of GB injection affect spleen DC activation. We found that *i*.*p*. or *intravenous* (*i*.*v*.) injection of GB showed almost similar effect on up-regulation of CD86, and MHC class I and II, whereas oral injection of GB did not induce up-regulation of these molecules (Fig 2C).

Next, to determine the effect of GB treatment on cytokine production, we injected 50 mg/kg GB to C57BL/6 mice for 12 or 24 hours and analyzed the levels of pro-inflammatory cytokines in splenocytes and blood serum. Administration of GB caused a marked increase in mRNA levels of IL-6, IL-12p40 and TNF-α in splenocytes 12 hours post-injection compared to PBS-treated control mice (Fig 2D). Serum levels of IL-6, IL-12p70 and TNF-α were also substantially increased by GB treatment 24 hours post-injection (Fig 2E). We next examined intracellular cytokine production in spleen DCs after 24 hours of GB treatment. As shown in Fig 2F, GB treatment led to marked increases in the percentage of IL-6-, IL-12- and TNF-α-producing spleen DCs. Therefore, systemic administration of GB induced activation of spleen DCs as indicated by up-regulation of co-stimulatory molecules and production of pro-inflammatory cytokines.

### GB Promotes Generation of Th1 and Tc1 Cells *in vivo*

**Fig 3:**
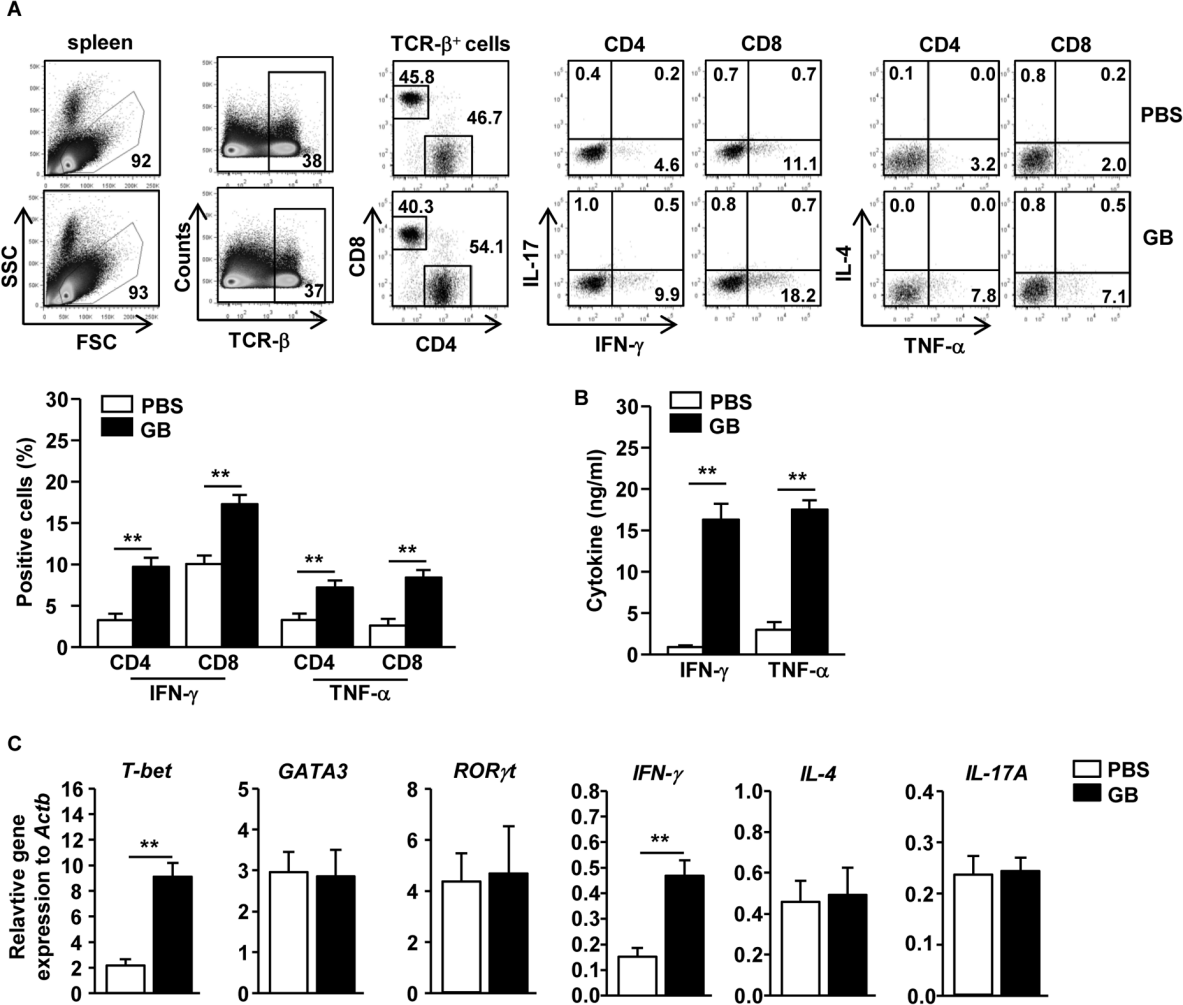
GB promotes IFN-γ-producing CD4 and CD8 T cells *in vivo*. C57BL/6 mice were injected *i*.*p*. with 50 mg/kg GB and 3 days later, injected again with same amount of GB for further 3 days. (A) Percentage of IFN-γ, IL-4, IL-17 and TNF-α positive cells within CD4 and CD8 T cells in spleen were assessed by flow cytometric analysis (Upper panel). Mean of IFN-γ and TNF-α positive cells in spleen (B) IFN-γ and TNF-α production levels in sera were measured by ELISA. (C) Expression levels of mRNA were measured from spleen 24 hours after GB injection. All data are representative of 6 samples from 3 independent experiments. **p < 0.*01*.

Since GB induced spleen DC activation, we next assessed whether GB-induced activation of spleen DCs can promote the generation of effector T cells, including Th1, Th2 and Th17 cells. C56BL/6 mice received *i*.*p*. injection of 50 mg/kg GB twice, 3 days apart, and were analyzed 3 days after the second injection. Intracellular production of IFN-γ and TNF-α, the critical cytokine for Th1 and Tc1 cells, were greatly increased in CD4 and CD8 T cells in GB-treated mice, whereas the percentages of IL-17- or IL-4-producing CD4 and CD8 T cells were not significantly increased (Fig 3A). Moreover, serum levels of IFN-γ and TNF-α were also markedly increased by GB treatment (Fig 3B). Furthermore, GB induced a marked increase in mRNA levels of IFN-γ and T-bet, the critical transcription factor for Th1 and Tc1 cells, in the spleen 24 hours after injection. However, the mRNA levels of GATA3 and RORγt, signature transcription factors for Th2 and Th17, and IL-4 and IL-17A were not changed by GB treatment (Fig 3C). These data indicate that GB treatment promotes Th1 and Tc1 responses *in vivo*.

### TLR4 and MyD88 Signaling Pathways are Essential for GB-induced DC activation

**Fig 4:**
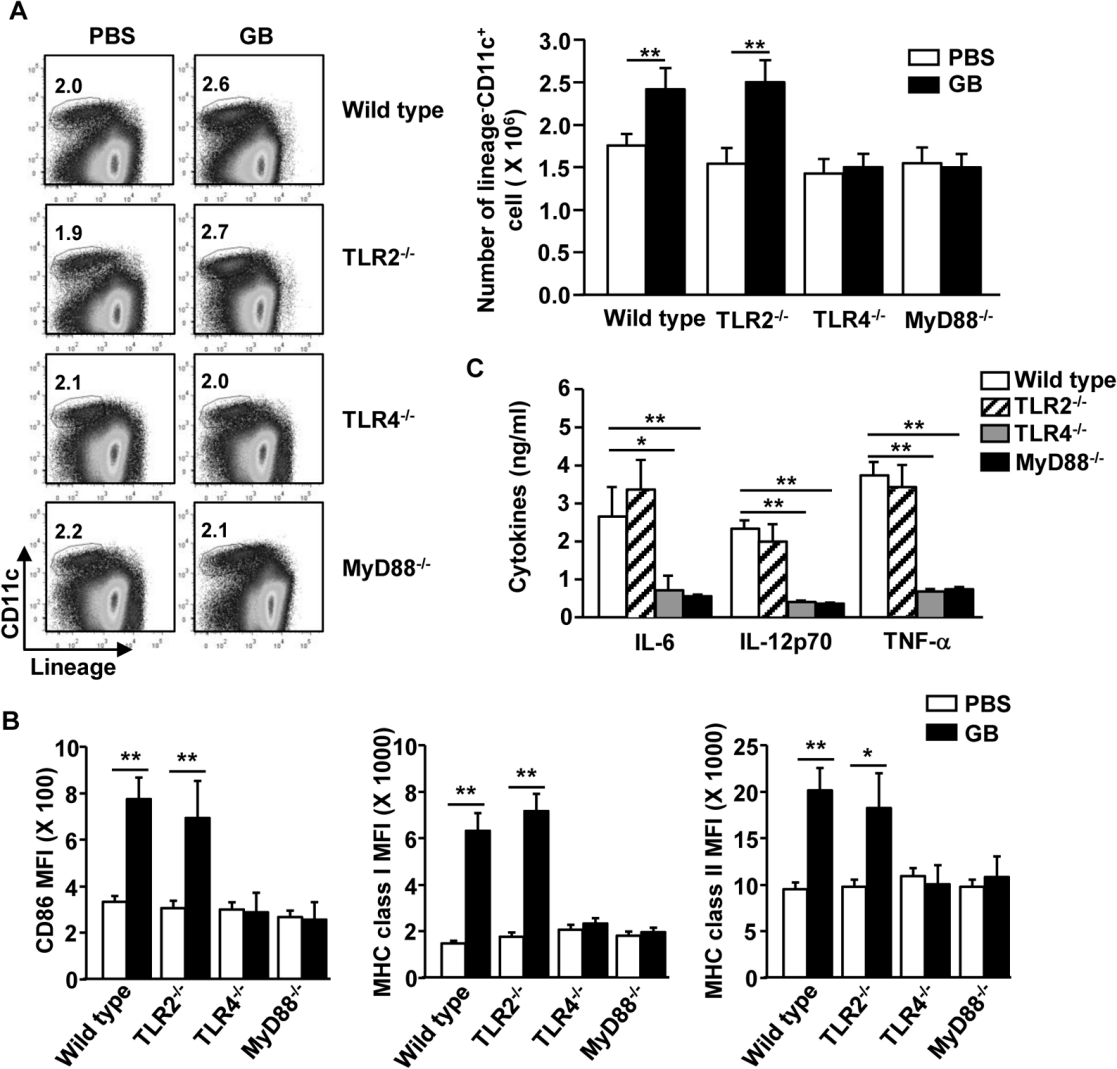
TLR4 and MyD88 singling pathway is essential for GB-induced DC activation. C57BL/6, TLR2^-/-^, TLR4^-/-^ or MyD88^-/-^ mice were injected *i*.*p*. with PBS or 50 mg/kg GB for 24 hours. (A) Percentage of lineage^-^CD11c^+^ spleen DCs was analyzed on a flow cytometry (left panels). Absolute cell number of spleen DCs within live cells were shown (right panel). (B) Expression level of CD86, MHC class I and MHC class II were measured by flow cytometry. (C) IL-6, IL-12p70 and TNF-α levels in sera were shown. All data are representative or the average of analyses of 6 samples from 3 independent experiments. *p < 0.*05*, **p < 0.*01*.

To further elucidate the mechanism by which GB promotes maturation of DCs, we examined the effect of GB in TLR2, TLR4 and MyD88 gene knockout mice. C57BL/6, TLR2^-/-^, TLR4^-/-^ or MyD88^-/-^ mice were injected *i*.*p*. with PBS or GB. As shown in Fig 4A, GB-induced increase in lineage^-^CD11c^+^ DC numbers was almost completely abrogated by TLR4- and MyD88-deficieny. Moreover, GB-induced up-regulation of CD86, MHC class I and MHC class II was also almost completely abolished by TLR4- and MyD88-deficiency (Fig 4B). In addition, GB-induced production of IL-6, IL-12 and TNF-α in serum was also severely impaired in TLR4^-/-^ and MyD88^-/-^ mice (Fig 4C). On the other hand, treatment of GB still induced an increase in lineage^-^CD11c^+^ DC numbers in TLR2^-/-^ mice (Fig 4A). Furthermore, GB-induced up-regulated of co-stimulatory molecules in spleen DCs and the production of pro-inflammatory cytokines were not impaired in TLR2^-/-^ mice (Fig 4B and 4C). Hence, these data indicate that GB-induced activation of spleen DCs is dependent on TLR4 and MyD88 signaling pathways but not TLR2 pathway.

### GB functions as an adjuvant to enhance antigen presentation and antigen specific T cell proliferation

**Fig 5:**
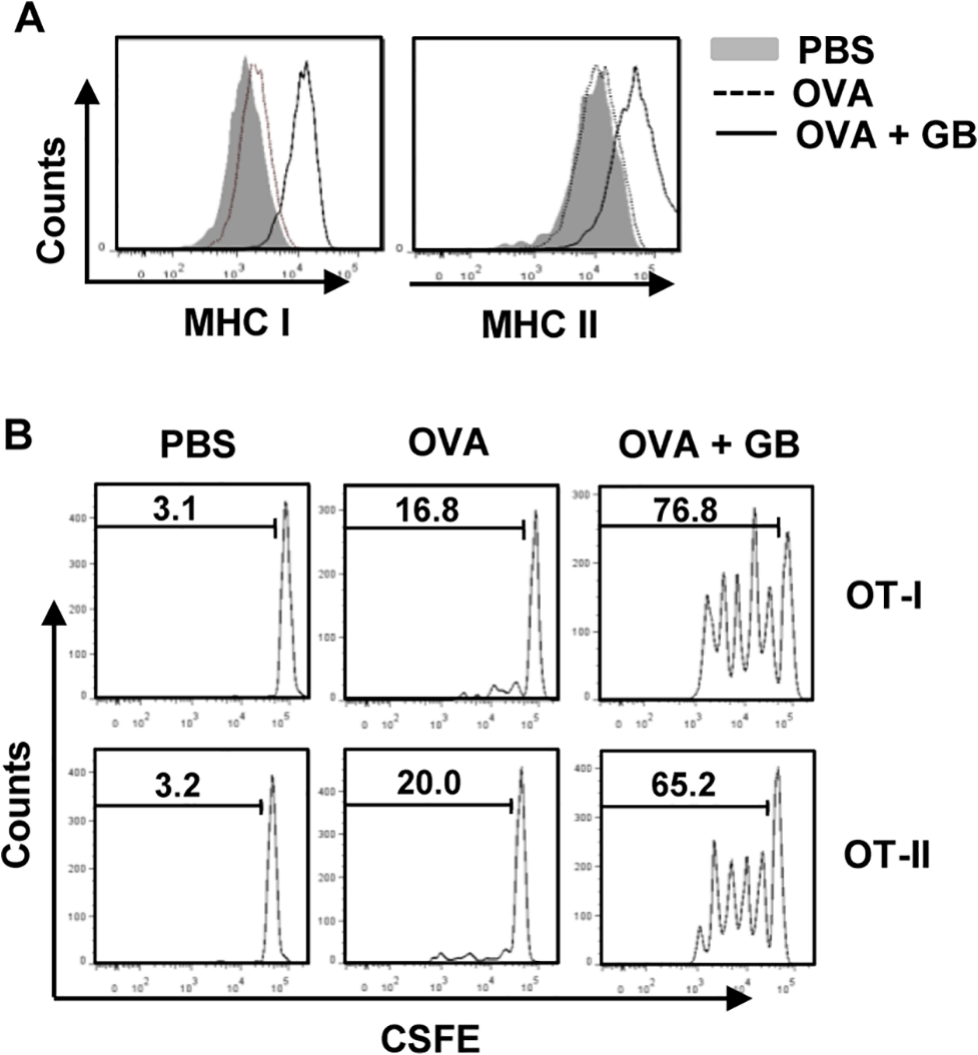
GB promotes antigen presentation and antigen-specific T cell proliferation *in vivo*. (A) Mice were injected with PBS, OVA or the combination of OVA and GB for 24 hours, and then expression levels of MHC class I and II were analyzed in lineage^-^CD11c^+^ DCs in spleen. (B) Purified CD8 T cell from OT-I or isolated CD4 T cell from OT-II were labeled with CFSE and transferred into CD45.1 congenic mice, and 24 hours later, mice were further treated with PBS, OVA or the combination of OVA and GB. After 3 day treatment, splenocytes were stained with CD45.2 and OT-I or OT-II cell proliferation was determined by CFSE dilution in CD45.2^+^ cells. All data are from analyses of 6 individual mice each group (2 mice per experiment, total 3 independent experiments).

To determine the adjuvant effect of GB in antigen-specific T cell response *in vivo*, we examined whether GB can promote antigen-presentation or cross presentation by DCs. C57BL/6 mice were injected with PBS, OVA or the combination of OVA and GB for 24 hours, and then measured for expression of MHC class I and II on spleen DCs. Spleen lineage^-^CD11c^+^ DCs substantially up-regulated surface expression of MHC class I and II molecules after treatment with the combination of OVA and GB, whereas those treated with OVA alone did not (Fig 5A). Next, to determine antigen-specific T cell proliferation, we transferred CFSE-labeled OT-I CD 8 T cells or OT-II CD4 T cells into CD45.1 congenic mice and 24 hours later, the mice received injection of PBS, OVA or the combination of OVA and GB. OT-I and OT-II T cells proliferation was robustly increased in mice immunized with the combination of OVA and GB compared to those in mice immunized with OVA alone, as determined by CFSE dilution after 3 day treatment (Fig 5B). These data demonstrated that GB functions as an adjuvant to enhance antigen presentation and antigen-specific CD4 and CD8 T cell activation.

### GB induces anti-cancer effect immune response

**Fig 6:**
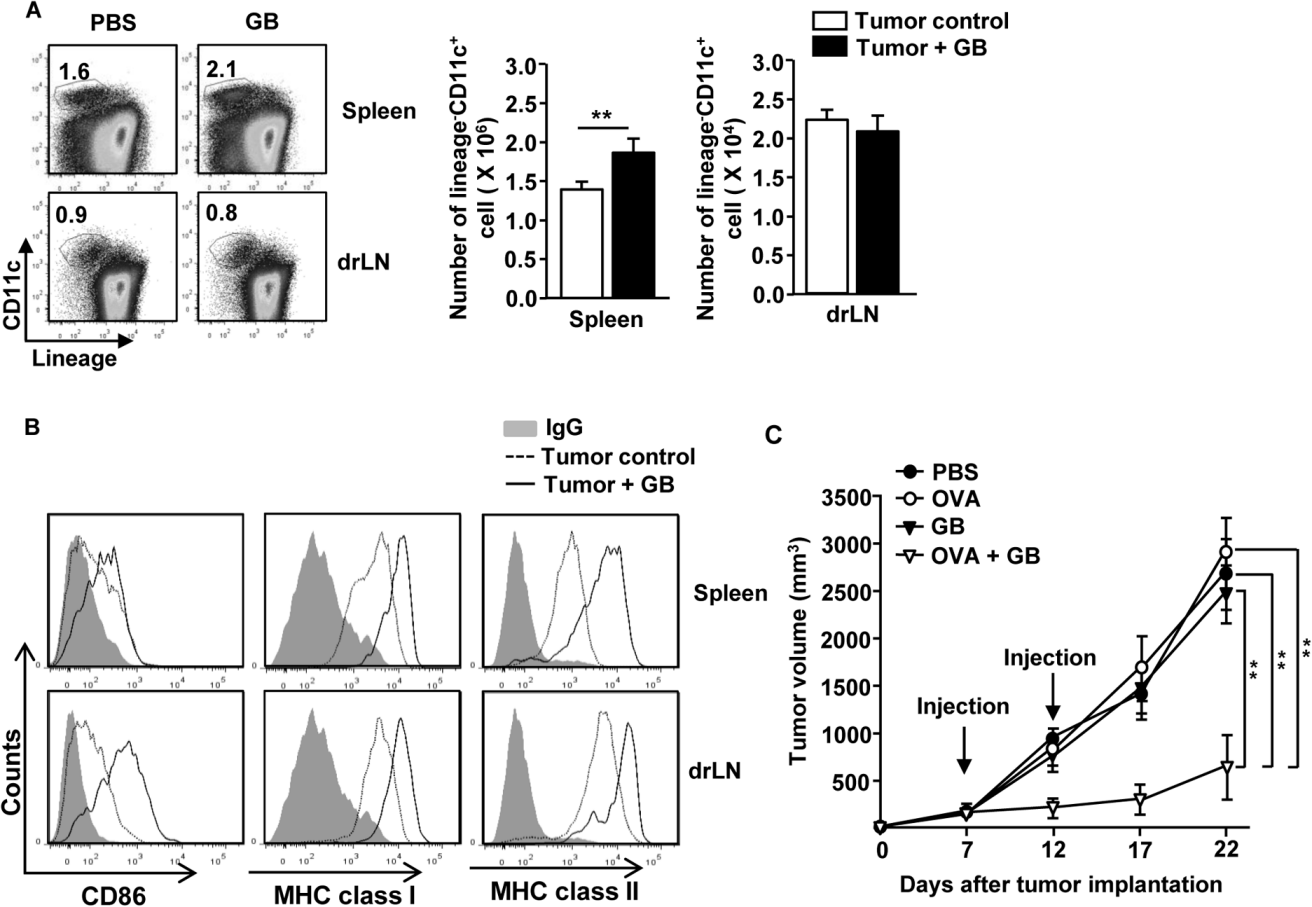
GB promotes anti-cancer activity by DC activation. C57BL/6 mice were injected *s*.*c*. with 1 X 10^6^ B16 cells. After 15 days, once tumors were well established, mice were treated with GB for 24 hours. (A) Percentage of lineage^-^CD11c^+^ DCs in spleen and drLN were analyzed on a flow cytometry (left panels). Absolute cell number of DCs in spleen and drLN were shown (right panel). **p < 0.*01*. (B) Expression level of CD86, MHC class I and MHC class II in spleen and drLN were measured by flow cytometry. (C) C57BL/6 mice were injected *s*.*c*. with 1 X 10^6^ B16 cells. Once tumors were well established on day 7, mice were received *i*.*p*. with PBS, OVA, GB or the combination of OVA and GB and 5 days later, treated with the same amount of OVA and GB again and tumor growth was measured. All data are representative of or the average of analyses of 6 independent samples (2 mice per experiment, total 3 independent experiments). **p < 0.*01*.

Since tumor environment suppresses immune cell activity [18], we examined whether GB can induce DC activation in tumor-bearing mice. C57BL/6 mice were injected *subcutaneously* (*s*.*c*.) with 1 X 10^6^ B16 melanoma cells. After 15 days, once tumors were well established, mice received GB injection and were analyzed for the activation of DCs in spleen and tumor draining lymph node (drLN) 24 hours later. Treatment of tumor-bearing mice with GB led to a substantial increase in the proportion and number of spleen DCs but not tumor drLN DCs (Fig 6A). Moreover, GB treatment of tumor-bearing mice induced up-regulation of CD80, MHC class I and II expression in spleen and tumor drLN DCs (Fig 6B).

Since GB can induce DC activation in the spleen and drLN of tumor-bearing mice, we next examined whether GB can promote therapeutic anti-cancer effect by enhancing Ag-specific immune responses. C57BL/6 mice were inoculated *s*.*c*. with 1 X 10^6^ B16-OVA tumor cells. After 7 days, once tumors were well established, mice were *i*.*p*.-injected with PBS, OVA, GB or the combination of OVA and GB, and 5 days later, injected again with the same amount of OVA and GB. B16-OVA tumor cell growth was almost completely inhibited by the combination of OVA and GB treatment, but not by GB or OVA treatment alone (Fig 6C). These data suggest that GB-induced antigen-specific activation of DCs can protect against tumor cell growth.

## Discussion

GR has been reported to prevent certain viral and bacterial infections [2, 19] and enhances immune activation as demonstrated by *in vitro* and *in vivo* studies [7, 20]. Although a variety of biological activities of ginseng extract from root, berry and leaf have been reported, its immune-related functions in the *in vivo* settings were not fully investigated. In this study, we demonstrated that GB has a stronger DC-activating effect than GR. Moreover, *in vivo* administration of GB induces maturation of spleen DCs and Th1 and Tc1 immune responses. OVA immunization in the presence of GB primed OVA-specific T cell proliferation, which together protected mice against the challenge of B16-OVA tumor cells. These data clearly demonstrate the adjuvant activity of GB, which mediated by DC activation.

In this study, we found that GB has a stronger BMDC-activating effect than GR. Consistent with our observation, GB showed better anti-hyperglycemic activity than GR when used at the same concentration [21]. The difference in their activities may result from different contents of ginsenosides. Ginseng extracts contain ginseng saponins (GS) and ginsenosides. Recent research has showed that ginsenoside can induce macrophage activation [22]. Importantly, GB has a higher content of total ginsenosides than GR, especially ginsenosides Re [21, 23, 24]. Moreover, ginsenosides Re and Rg1, which are major contents of GR, have been shown to activate TLR4 signaling pathway [25, 26]. We also showed that GR-induced spleen DC maturation is dependent on TLR4 signaling pathway. We thus hypothesize that ginsenosides, such as Re and Rg1, are the major components that induced spleen DC maturation, and that the higher contents of ginsenosides in GB account for its higher potency of effect compared to GR. Further research will be required to test this hypothesis and determine whether specific ginsenosides purified from GB can promote activation of spleen DCs and TLR4 signaling pathway.

We also found that oral administration of GB does not induce spleen DC activation, although ginseng products are mostly used as edible medicine. It has been shown that oral administration of LPS can activate intestinal B-1 cells and protect against bacterial in colon [27–29], but cannot induce systemic immune responses [28, 30]. Similarly, *i*.*p*. or *i*.*v*. administration of GB may be required for inducing systemic immune activation. It will also be important to determine whether oral administration of GB can induce intestinal immune activation and exert protective effect against intestinal bacterial infection.

DCs can be activated by pathogen components through pattern recognition receptors (PRRs) [31]. Recent studies have shown that various types of natural products can activate DCs through both PRRs, such as TLRs and scavenger receptors (SRs) [32–35]. Moreover, many lines of research showed that TLR ligand-induced DC maturation requirs MyD88 signaling pathway [16, 35, 36]. We also found that GB-induced DC activation is dependent on TLR4 and MyD88 signaling pathways. These data suggest that ginsenosides in GB may stimulate TLRs to induce spleen DC activation. To determine this, our future studies will investigate whether purified ginsenosides from GB can induce spleen DC activation.

The development of therapeutic cancer vaccines is a challenging task. For therapeutic vaccines to be effective, they must circumvent regulatory mechanisms that limit the activation of DCs and expansion of CD4 and CD8 T cells [18, 33]. Thus, the need to enhance immunogenicity of peptide vaccines is paramount. An effective vaccine adjuvant should boost cell-mediated antigen-specific immune responses in order to effectively eliminate pathogens [13, 14]. However, since tumor environment suppresses immune activities, many vaccine adjuvants cannot induce full activation of immune responses against specific tumor Ags. In this study, our findings indicate that GB exhibits an adjuvant activity of priming T cell responses to the soluble OVA Ag. Moreover, GB induces the activation of spleen and tumor drLN DCs in tumor-bearing mice. Finally, administration of GB plus OVA to tumor-bearing mice successfully inhibits B16-OVA tumor cell growth, suggesting that the activation of spleen and tumor drLN DCs by GB may in turn enhance OVA-specific Th1 and CTL responses, which consequently inhibit B16-OVA tumor cell growth.

In conclusion, our results provide evidence that the GB is a novel therapeutic tumor vaccine adjuvant, which can stimulate DC activation, Th1 and Tc1 immune responses, and Ag-specific T cell proliferation to inhibit tumor cell growth. The immune-activating effect of GB will make it a promising candidate in the translational and clinical research of tumor vaccines.

## References

[1] Ramberg JE, Nelson ED, Sinnott RA. Immunomodulatory dietary polysaccharides: a systematic review of the literature. Nutrition journal. 2010;9:54 10.1186/1475-2891-9-54 21087484 PMC2998446

[2] Blumenthal M. Asian ginseng: potential therapeutic uses. Advance for nurse practitioners. 2001;9(2):26–8, 33. . 12416051

[3] Zhang G, Huihua G, Yi L. Stability of halophilic proteins: from dipeptide attributes to discrimination classifier. International journal of biological macromolecules. 2013;53:1–6. 10.1016/j.ijbiomac.2012.10.031 . 23142140

[4] Byeon SE, Lee J, Kim JH, Yang WS, Kwak YS, Kim SY, et al Molecular mechanism of macrophage activation by red ginseng acidic polysaccharide from Korean red ginseng. Mediators of inflammation. 2012;2012:732860 10.1155/2012/732860 22474399 PMC3306998

[5] Park EY, Kim HJ, Kim YK, Park SU, Choi JE, Cha JY, et al Increase in Insulin Secretion Induced by Panax ginseng Berry Extracts Contributes to the Amelioration of Hyperglycemia in Streptozotocininduced Diabetic Mice. Journal of ginseng research. 2012;36(2):153–60. 10.5142/jgr.2012.36.2.153 23717115 PMC3659577

[6] Tran TL, Kim YR, Yang JL, Oh DR, Dao TT, Oh WK. Dammarane triterpenes from the leaves of Panax ginseng enhance cellular immunity. Bioorganic & medicinal chemistry. 2014;22(1):499–504. 10.1016/j.bmc.2013.11.002 . 24290061

[7] Zhai L, Li Y, Wang W, Wang Y, Hu S. Effect of oral administration of ginseng stem-and-leaf saponins (GSLS) on the immune responses to Newcastle disease vaccine in chickens. Vaccine. 2011;29(31):5007–14. 10.1016/j.vaccine.2011.04.097 . 21569814

[8] Banchereau J, Palucka AK. Dendritic cells as therapeutic vaccines against cancer. Nature reviews Immunology. 2005;5(4):296–306. 10.1038/nri1592 . 15803149

[9] Banchereau J, Steinman RM. Dendritic cells and the control of immunity. Nature. 1998;392(6673):245–52. 10.1038/32588 . 9521319

[10] Pooley JL, Heath WR, Shortman K. Cutting edge: intravenous soluble antigen is presented to CD4 T cells by CD8- dendritic cells, but cross-presented to CD8 T cells by CD8+ dendritic cells. Journal of immunology. 2001;166(9):5327–30. . 11313367 10.4049/jimmunol.166.9.5327

[11] Shortman K, Heath WR. The CD8+ dendritic cell subset. Immunological reviews. 2010;234(1):18–31. 10.1111/j.0105-2896.2009.00870.x . 20193009

[12] Villadangos JA, Schnorrer P. Intrinsic and cooperative antigen-presenting functions of dendritic-cell subsets in vivo. Nature reviews Immunology. 2007;7(7):543–55. 10.1038/nri2103 . 17589544

[13] Dubensky TW Jr, Reed SG. Adjuvants for cancer vaccines. Seminars in immunology. 2010;22(3):155–61. 10.1016/j.smim.2010.04.007 . 20488726

[14] Coffman RL, Sher A, Seder RA. Vaccine adjuvants: putting innate immunity to work. Immunity. 2010;33(4):492–503. 10.1016/j.immuni.2010.10.002 21029960 PMC3420356

[15] Kim CK, Cho DH, Lee KS, Lee DK, Park CW, Kim WG, et al Ginseng Berry Extract Prevents Atherogenesis via Anti-Inflammatory Action by Upregulating Phase II Gene Expression. Evidence-based complementary and alternative medicine: eCAM. 2012;2012:490301 10.1155/2012/490301 23243449 PMC3519292

[16] Zhang W, Du JY, Jiang Z, Okimura T, Oda T, Yu Q, et al Ascophyllan purified from Ascophyllum nodosum induces Th1 and Tc1 immune responses by promoting dendritic cell maturation. Marine drugs. 2014;12(7):4148–64. 10.3390/md12074148 25026264 PMC4113820

[17] Wang Z, Meng J, Xia Y, Meng Y, Du L, Zhang Z, et al Maturation of murine bone marrow dendritic cells induced by acidic Ginseng polysaccharides. International journal of biological macromolecules. 2013;53:93–100. 10.1016/j.ijbiomac.2012.11.009 . 23164755

[18] Zou W. Regulatory T cells, tumour immunity and immunotherapy. Nature reviews Immunology. 2006;6(4):295–307. 10.1038/nri1806 . 16557261

[19] Lee JS, Ko EJ, Hwang HS, Lee YN, Kwon YM, Kim MC, et al Antiviral activity of ginseng extract against respiratory syncytial virus infection. International journal of molecular medicine. 2014;34(1):183–90. 10.3892/ijmm.2014.1750 24756136 PMC4072342

[20] Kimura M, Waki I, Chujo T, Kikuchi T, Hiyama C, Yamazaki K, et al Effects of hypoglycemic components in ginseng radix on blood insulin level in alloxan diabetic mice and on insulin release from perfused rat pancreas. Journal of pharmacobio-dynamics. 1981;4(6):410–7. . 7026762 10.1248/bpb1978.4.410

[21] Dey L, Xie JT, Wang A, Wu J, Maleckar SA, Yuan CS. Anti-hyperglycemic effects of ginseng: comparison between root and berry. Phytomedicine: international journal of phytotherapy and phytopharmacology. 2003;10(6–7):600–5. 10.1078/094471103322331908 . 13678250

[22] Wang Y, Liu Y, Zhang XY, Xu LH, Ouyang DY, Liu KP, et al Ginsenoside Rg1 regulates innate immune responses in macrophages through differentially modulating the NF-kappaB and PI3K/Akt/mTOR pathways. International immunopharmacology. 2014;23(1):77–84. 10.1016/j.intimp.2014.07.028 . 25179784

[23] Choi YD, Park CW, Jang J, Kim SH, Jeon HY, Kim WG, et al Effects of Korean ginseng berry extract on sexual function in men with erectile dysfunction: a multicenter, placebo-controlled, double-blind clinical study. International journal of impotence research. 2013;25(2):45–50. 10.1038/ijir.2012.45 . 23254461

[24] Cho KS, Park CW, Kim CK, Jeon HY, Kim WG, Lee SJ, et al Effects of Korean ginseng berry extract (GB0710) on penile erection: evidence from in vitro and in vivo studies. Asian journal of andrology. 2013;15(4):503–7. 10.1038/aja.2013.49 23708462 PMC3739245

[25] Su F, Yuan L, Zhang L, Hu S. Ginsenosides Rg1 and Re act as adjuvant via TLR4 signaling pathway. Vaccine. 2012;30(27):4106–12. 10.1016/j.vaccine.2012.03.052 . 22472794

[26] Zhao BS, Liu Y, Gao XY, Zhai HQ, Guo JY, Wang XY. Effects of ginsenoside Rg1 on the expression of toll-like receptor 3, 4 and their signalling transduction factors in the NG108-15 murine neuroglial cell line. Molecules. 2014;19(10):16925–36. 10.3390/molecules191016925 . 25340298 PMC6271333

[27] Chen LW, Chang WJ, Chen PH, Hsu CM. Commensal microflora induce host defense and decrease bacterial translocation in burn mice through toll-like receptor 4. Journal of biomedical science. 2010;17:48 10.1186/1423-0127-17-48 20540783 PMC2901327

[28] Inagawa H, Kohchi C, Soma G. Oral administration of lipopolysaccharides for the prevention of various diseases: benefit and usefulness. Anticancer research. 2011;31(7):2431–6. . 21873155

[29] Murakami M, Tsubata T, Shinkura R, Nisitani S, Okamoto M, Yoshioka H, et al Oral administration of lipopolysaccharides activates B-1 cells in the peritoneal cavity and lamina propria of the gut and induces autoimmune symptoms in an autoantibody transgenic mouse. The Journal of experimental medicine. 1994;180(1):111–21. 8006578 10.1084/jem.180.1.111 PMC2191544

[30] Oketani K, Inoue T, Murakami M. Effect of E3040, an inhibitor of 5-lipoxygenase and thromboxane synthase, on rat bowel damage induced by lipopolysaccharide. European journal of pharmacology. 2001;427(2):159–66. . 11557269 10.1016/s0014-2999(01)01234-1

[31] Gordon S. Pattern recognition receptors: doubling up for the innate immune response. Cell. 2002;111(7):927–30. . 12507420 10.1016/s0092-8674(02)01201-1

[32] Jin JO, Park HY, Xu Q, Park JI, Zvyagintseva T, Stonik VA, et al Ligand of scavenger receptor class A indirectly induces maturation of human blood dendritic cells via production of tumor necrosis factor-alpha. Blood. 2009;113(23):5839–47. 10.1182/blood-2008-10-184796 . 19351958

[33] Jin JO, Zhang W, Du JY, Wong KW, Oda T, Yu Q. Fucoidan can function as an adjuvant in vivo to enhance dendritic cell maturation and function and promote antigen-specific T cell immune responses. PloS one. 2014;9(6):e99396 10.1371/journal.pone.0099396 24911024 PMC4049775

[34] Lin CY, Lu MC, Su JH, Chu CL, Shiuan D, Weng CF, et al Immunomodulatory effect of marine cembrane-type diterpenoids on dendritic cells. Marine drugs. 2013;11(4):1336–50. 10.3390/md11041336 23609581 PMC3705408

[35] Calzas C, Goyette-Desjardins G, Lemire P, Gagnon F, Lachance C, Van Calsteren MR, et al Group B Streptococcus and Streptococcus suis capsular polysaccharides induce chemokine production by dendritic cells via Toll-like receptor 2- and MyD88-dependent and-independent pathways. Infection and immunity. 2013;81(9):3106–18. 10.1128/IAI.00113-13 23774593 PMC3754219

[36] Ochi A, Nguyen AH, Bedrosian AS, Mushlin HM, Zarbakhsh S, Barilla R, et al MyD88 inhibition amplifies dendritic cell capacity to promote pancreatic carcinogenesis via Th2 cells. The Journal of experimental medicine. 2012;209(9):1671–87. 10.1084/jem.20111706 22908323 PMC3428946

